# Application of High-Resolution Remote-Sensing Data for Land Use Land Cover Mapping of University Campus

**DOI:** 10.1155/2021/5519011

**Published:** 2021-07-30

**Authors:** Irwan Ary Dharmawan, Muhammad Ario Eko Rahadianto, Edward Henry, Cipta Endyana, Muhammad Aufaristama

**Affiliations:** ^1^Department of Geophysics, Faculty of Mathematics and Natural Sciences, Universitas Padjadjaran, Jl. Raya Bandung Sumedang km. 21, Jatinangor, Sumedang 45363, Jawa Barat, Indonesia; ^2^Directorate of Facilities, Infrastructures and Asset Management, Universitas Padjadjaran, Jl. Raya Bandung Sumedang km. 21, Jatinangor, Sumedang 45363, Jawa Barat, Indonesia; ^3^Faculty of Geological Engineering, Universitas Padjadjaran, Jl. Raya Bandung Sumedang km. 21, Jatinangor, Sumedang 45363, Jawa Barat, Indonesia; ^4^University of Twente, Faculty for Geo-Information Science and Earth Observation (ITC), P.O. Box 6, 7500 AA Enschede, Netherlands

## Abstract

The study of Land Use Land Cover (LULC) is essential to understanding how land has been altered in recent years and what has caused the processes behind the change. This is significant for the future development of the area, particularly on the campus of the Universitas Padjadjaran Jatinangor. The purpose of this study was to apply remote-sensing techniques to map a university campus and vicinity by comparing the area of urban green space (UGS) and floor area ratios (FARs) of the campus in 2015 and 2017. Additionally, surface runoff analysis was also conducted. For our research, we used WorldView-2's high-resolution satellite imagery with a resolution of 0.46 m in the Universitas Padjadjaran (Padjadjaran University, or Unpad) Jatinangor campus, Jawa Barat, Indonesia. Our approach was to interpret the imagery by running the normalized difference vegetation index (NDVI) to distinguish UGS and FAR and using digital elevation model (DEM) interferometric synthetic aperture radar (SAR) data with hydrologic analysis to identify the direction of surface runoff. The results obtained are as follows: the UGS remained more extensive compared with FAR, but the difference decreased over time owing to infrastructure development. Surface runoff has tended to flow toward the southeast in direct relation to the slope configuration.

## 1. Introduction

According to the 2019 QS Star ranking, Universitas Padjadjaran (Unpad) is among the top five universities in Indonesia [1]. Unpad has ∼40,000 students, which are distributed in 16 faculties. As a developing university, Unpad constantly grows the amount of infrastructure and facilities to support students' learning process, to support research staff activities, and to achieve world-class university status. Unpad has a main campus located in Jatinangor, Jawa Barat, Indonesia, with an area of ∼178 ha.

As a state university, Unpad must have a stake in the development and preservation of urban green space (UGS). The Unpad campus also has infrastructure problems that need to be studied and addressed. One of them is based on the strict Regional Regulation (Perda) of Sumedang Regency no. 2 of 2012 concerning regional spatial planning, which states that the ratio of UGS to floor area ratios (FARs) for any business activity, large or small, must meet or exceed 60%:40% [2]. The need for Unpad to add other buildings requires consideration of these regulations.

Another problem is the impact of campus land-use and land-cover (LULC) changes that affect surface runoff. To prevent flooding during the rainy season, further analysis of surface runoff is needed to maintain and enhance the capability of the land to use or absorb standing water [3], which can be a source of water reserved for use by the current Unpad campus.

LULC usually refers to the classification of human activities and natural features in the landscape based on the established scientific and statistical methods of material analysis within a specific time frame [4]. LULC mapping has a wide range of uses such as urban planning [5, 6], hazard mitigation [7, 8], ecological studies [9], and environmental studies [10, 11]. Using a similar approach, we can implement LULC analysis for the university campus. However, it is quite difficult to map LULC using images with moderate resolution in a small coverage area such as a campus. Therefore, to map a small area in detail, it is necessary to use high-resolution images.

Remote sensing and GIS are important tools for studying LULC changing and integrating the associated driving factors for deriving useful outputs. Remote sensing combines science and art to obtain data and information about objects that exist on the Earth's surface by using tools that are not directly related to the object being studied [12]. Remote-sensing techniques have been widely used in many applications, such as for urban-planning purposes [13–16]. While most of the urban research has been done on large cities or metropolitan areas such as a city in Asia [16–19], in America [20], and worldwide [21]; in a few cases, it is applied to specific areas such as a university campus or school. However, some previous researchers used only low- to moderate-resolution satellite data for LULC analysis; for instance, traditional aerial photography [22], Landsat with 30 m resolution [19, 22], and the combination of ASTER and Landsat with a different resolution [18]. On the other hand, Weng [14] successfully discussed different types of data and methods that can be used to map LULC changes.

The purpose of our research is in line with Unpad's mission to reach a sustainable campus. In our research, we used high-resolution multitemporal imagery from the WorldView-2 DigitalGlobe satellite for campus evaluation, planning, and development. In addition, we field validated the resulting data set.

## 2. Remote Sensing for Regional Mapping

### 2.1. WorldView-2 Satellite Sensor and Digital Elevation Model (DEM)

The WorldView-2 satellite was launched in October 2009. The remote-sensing imagery produced has a high spatial resolution and also has a more-complete spectral resolution than the previous DigitalGlobe imagery sensor. The spatial resolution of WorldView-2 products is 0.46–0.5 m for panchromatic imagery and 1.84 m for eight-channel multispectral imagery. Channels capture a range or spectrum of wavelengths (Table 1). These specifications are quite adequate for spatial analysis of natural resources and the environment. High-resolution WorldView-2 (WV-2) images acquired on June 14th, 2015, and September 17th, 2017, with less than 20% cloud cover were used in this study.

The specific or best-use application of each of the eight channels [23] recorded in multispectral imagery is as follows: Coastal blue for vegetation analysis, bathymetry, atmospheric correction; Blue for vegetative analysis, bathymetry, atmospheric correction; Green for analysis of healthy plants, plant strength, categorization of plants assisted by yellow channels; Yellow for detection of yellowness of vegetation, both on land and in water; Red for distinguishing vegetation, classification of land, roads, geological features; Red edge for measuring plant health and classifying plants; Near infrared 1 for estimation of water content and plants biomass, separation of waterbodies from vegetation, identification of vegetation types and soil types; and Near infrared 2 for same as near infrared 1 but less influenced by the atmosphere.

To identify built-up land features through unsupervised classification, Masek et al. established a technique based on a normalized difference vegetation index (NDVI) [24]. Instead of other building indices such as NDBI, in this research, we used the NDVI for building assessment due to limitation of SWIR (shortwave infrared) bands in our WorldView data sets. This approach can be an alternative when a spectral limitation exists [25]. Moreover, the use of WV-2 image for detailed mapping is expanding due to its very high spatial resolution.

Several studies have attempted to extract classification and feature detection from remotely sensed images with very high spatial resolution [26–29], given land classification limitations such as time-consuming field surveys, accurate, and timely.

Also, one of the remote-sensing products is the DEM, which is a digital model of a topographic surface or terrain. More specifically, a DEM is a digital data set portraying the geometry of the shape of part of the Earth's surface and consists of a set of points sampled from the surface by an algorithm that defines the surface in terms of coordinates [30]. The term DEM was popularized by the U.S. Geological Survey (USGS). DEMs are usually developed from remote-sensing data, but they can be developed from field-survey data. They are often used in geographic information systems.

### 2.2. Land Use and Land Cover (LULC)

Land cover is the condition of the observed biophysical appearance of the Earth's surface. Land cover can be grouped into vegetated and nonvegetated areas [31]. Vegetated-areas classification includes irrigated rice fields, rainfed rice fields, tidal rice fields, plantations, mixed crops, dryland forests, wetland forests, shrubs, savanna, imperata grasslands, and swamp grass. Nonvegetated-areas classification includes open land on a caldera, lava, stretch of beach sand, beach shoals, sand dunes, riverbanks, built land, settlement, industrial buildings, road network, railroad network, high-voltage electricity network, domestic/international airports, land not built, mining, landfill deposits, lakes, rivers, irrigation channels, and coral reefs.

Supported by land-cover information, further analysis yields land-use information. Land use includes all types by humans, including land use for agriculture, office buildings, sports fields, houses, businesses, hospitals, etc. [32]. Land has an important function in human life. However, changes in land use from nonbuilt to built can reduce the ability of land to absorb rainwater. In this study, land use was classified as UGS or FAR, in accordance with Perda [2].

The classification UGS is an open, extensive clustered area or path used for plants, both growing naturally and intentionally planted. It is divided into two types, public and private green space. Public UGSs are owned and managed by the city or regency regional government for the benefit of the general community; examples include city parks, urban forests and green belts, and land along rivers and railroads. In contrast, private UGSs belong to certain institutions or individuals, and their use is limited to certain owners or permitted people or groups; examples include private yards or gardens as well as the grounds around community-owned buildings that have private plantings.

The FAR refers to the percentage that the ground-floor area of a building may occupy compared with the available land area. To be included in the FAR, a structure must consist of a sizable section of roofed-over rooms that have walls of height more than 1.2 m or it must be within the definition of projected buildings, which are open spaces at ground level that are under the building [33].

### 2.3. Surface-Runoff Direction

Surface runoff is water flowing on the ground surface owing to impermeable ground or soils having reached full infiltration capacity. This runoff flows downslope toward and into a basin or depression in the land surface [34]. After such filling, the water further flows freely over the ground. DEM analysis can be used to map water-flow direction and water accumulation. This hydrologic approach to map surface runoff is based entirely on topographic differences.

## 3. Methodology

### 3.1. Area of Study

**Figure 1:**
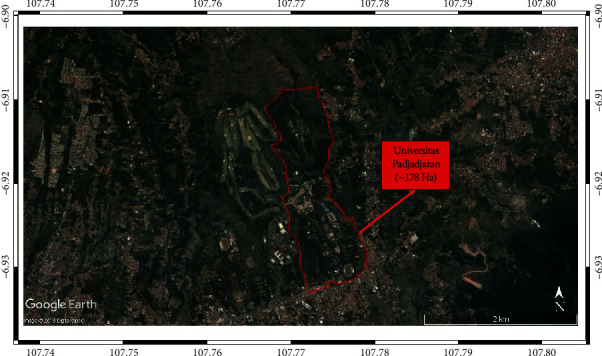
Study area [35], showing boundaries of the 178 ha Unpad campus.

The Unpad Jatinangor campus is in Jatinangor District, Sumedang Regency, Jawa Barat, Indonesia. The geographic location of the study area (the campus and vicinity) is south and east of 107°46′28″ S and 6°55′33″E with altitudes of ∼725–810 m above sea level. The Unpad comprises 16 faculties spread throughout the ∼178 ha campus (see Figure 1), which has extensive areas of vegetation, including an arboretum open to visitors and plantations that provide a learning environment for student discussions. Lake Cekdam on the Unpad campus, which is near the university's arboretum, has an area of ∼1 ha and serves as a reservoir to collect rainwater.

### 3.2. NDVI

Remote sensing of vegetation depends on unique spectral characteristics associated with biophysical processes that occur within plants. For instance, leaves absorb and disperse radiation, and the level of absorption or scattering is a function of the wavelength [29]. Green leaves absorb radiation from the visible light channel (blue-red end of the spectrum) and reflect radiation from the near-infrared channel. Strong absorption in the red channel has been correlated with biomass production. Reflection associated with the near-infrared channel is related to the leaves' internal light scattering and green-leaf density, which increases with photosynthetic activity [36]. This transformation of the NDVI combines the band ratio and channel-reduction methods [37] involving the red channel absorbed by green leaves and near-infrared channels reflected by green leaves. The NDVI is often used in analysis of LULC changes [38–42]. The equation to calculate the NDVI is

(1) $$\begin{array}{c} \text{NDVI}=\;\frac{\rho_{\text{nir}}-\rho_{\text{red}}}{\rho_{\text{nir}}+\rho_{\text{red}}}, \end{array}$$

where *ρ*_nir_ is the reflectance received by the near-infrared-channel sensor (spectrum of wavelengths in the range 840–1060 nm), and *ρ*_red_ is the reflection intensity received by the red-channel sensor (spectrum of wavelengths in the range 630–690 nm). The NDVI value is close to −1 if it does not detect the presence of vegetation (buildings, water, snow, etc.) but is 1 if it detects vegetation. The denser the vegetation is, the closer the value of NDVI is to 1 [43, 44].

### 3.3. Land-Cover Analysis

To compare vegetated and nonvegetated areas of the Unpad campus, we performed land-cover analysis using NDVI transformation processing, which can distinguish areas of vegetation from those without vegetation (buildings, water, bare fields, etc.). We interpreted the NDVI results against RGB (red-green-blue color gamut) images to categorize the index values according to how they correlate with the type of land cover. We classified the land cover into five types: water, buildings, empty land, low/sparse vegetation, and high/dense vegetation. Next, we applied histogram analyses of the pixel values of the NDVI images to calculate areal extent and percentage of vegetated and nonvegetated land cover within the Unpad campus boundary. For the total land area, as defined by the National Standardization Agency for 2010 [45], we added vegetated and nonvegetated land and applied equations (2) and (3) to derive the overall percentages of each (%*A*_veg_ and %*A*_nonveg_):

(2) $$\begin{array}{c} \%A_{\text{veg}}=\frac{A_{\text{veg}}}{A_{\text{tot}}}\times 100\%, \end{array}$$

(3) $$\begin{array}{c} \%A_{\text{nonveg}}=\frac{A_{\text{nonveg}}}{A_{\text{tot}}}\times 100\%, \end{array}$$

assuming

(4) $$\begin{array}{c} A_{\text{tot}}=A_{\text{veg}}+A_{\text{nonveg}}, \end{array}$$

where *A*_tot_, the total area, is 178 ha; *A*_veg_ is the area of vegetated land cover; and *A*_nonveg_ is the area of nonvegetated land cover. We also compared the 2015 and 2017 imagery of the Unpad campus to determine land-cover change.

In 2015, Unpad obtained an infrastructure grant from the Ministry of Higher Education for the establishment of buildings and facilities of the Nursing Faculty. Furthermore, the grant is also being used for the development of the Science Technopark buildings, which focuses on incubating and downstream research from Unpad researchers. In addition, in 2018, Unpad obtained a long contract on the establishment of animal hospitals, student centers, and educational hospitals. According to the regulation, the calculation of the ratio of UGS to FAR in every infrastructure establishment must be recalculated.

### 3.4. Land-Use Analysis

To validate our results, we conducted analyses of UGS and FAR in several stages—ground truth, categorization of UGS and FAR, and comparison of UGS and FAR in 2015 versus 2017. The UGS category includes both land deliberately planted (gardens, parks, etc.) and land covered by natural plant growth. In contrast, the FAR category consists of land that is covered over by roads, buildings, or other manmade structures. Ground truth conducted to minimize errors in image interpretation was performed by marking locations that are not clearly visible on a digital map (such as areas covered by dense tall plants). Such checks were conducted periodically; the first such field inspection was done on November 11, 2018, and the result is shown in Table 2. To classify UGS versus FAR (done by using QGIS software's shape tool), we considered the results of field checks, image color analysis, and NDVI transformations. This type of data was stored as shapefiles (using ∗.shp format) so that it can be opened in other GIS software. We also ran extensive calculations on the UGS and FAR data and compared the 2015 and 2017 results to assess their relative decrease and increase. On the basis of designations by the Perda [2], we determined total land area as the sum of the UGS and FAR areas. Accordingly, we used equations (5) and (6) to derive the overall percentages of each (%*A*_UGS_ and %*A*_FAR_):

(5) $$\begin{array}{c} \%A_{\text{UGS}}=\frac{A_{\text{UGS}}}{A_{\text{tot}}}\times 100\%, \end{array}$$

(6) $$\begin{array}{c} \%A_{\text{FAR}}=\frac{A_{\text{FAR}}}{A_{\text{tot}}}\times 100\%, \end{array}$$

assuming

(7) $$\begin{array}{c} A_{\text{tot}}=A_{\text{UGS}}+A_{\text{FAR}}, \end{array}$$

where *A*_tot_ is the total area (UGS and FAR combined), *A*_UGS_ is the total area of just UGS, and *A*_FAR_ is the total area of just FAR.

### 3.5. Surface-Runoff Analysis

To determine trends in the various directions of water flow and also to gather information on surface-water accumulation, we used DEM data to do surface-runoff analysis. The DEM data that we used were corrected by applying the fill sinks algorithm [46]. Such analysis is done in successive stages, the first for flow direction and the second for flow accumulation. The initial flow-direction analysis is done for each pixel, for which we used Saga GIS software's D8 or eight-direction, analytical method. The results are in the form of raster map data with separate values for each pixel based on the flow-direction tendencies generated from the DEM data.

**Figure 2:**
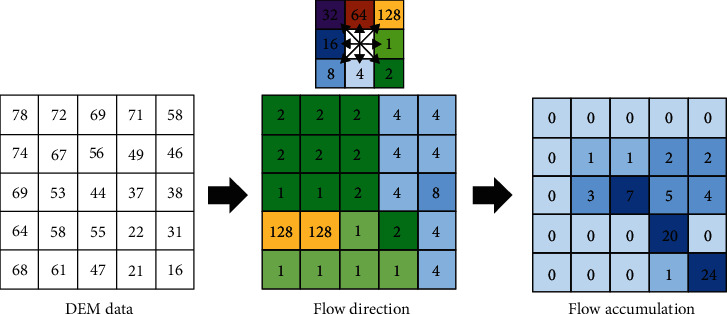
Schematic of the algorithm for flow direction and accumulation. The diagram in upper middle shows eight flow directions (and their labels).

The principle of flow analysis using the D8 algorithm (see Figure 2) is to identify and select the most-significant decrease for each cell as the flow direction. For each pixel, the algorithm finds the greatest difference in value compared to surrounding pixels to assign one of eight flow-direction values: 1 for east, 2 for southeast, 4 for south, 8 for southwest, 16 for west, 32 for northwest, 64 for north, and 128 for northeast.

Flow accumulation is derived by summing the flow directions of the cells flowing to a calculated cell (called weight). The higher a cell's calculated weight, the greater the possibility of flow accumulation in the cell. Flow accumulation may be associated with river flow. Pixel results from applying the flow-direction algorithm are then retranslated according to the number of surrounding pixels with incoming flow. Pixel values are large if there are many such pixels but are small or zero if the adjacent pixels receive low or no flow.

## 4. Result and Discussion

### 4.1. Land Cover

**Figure 3:**
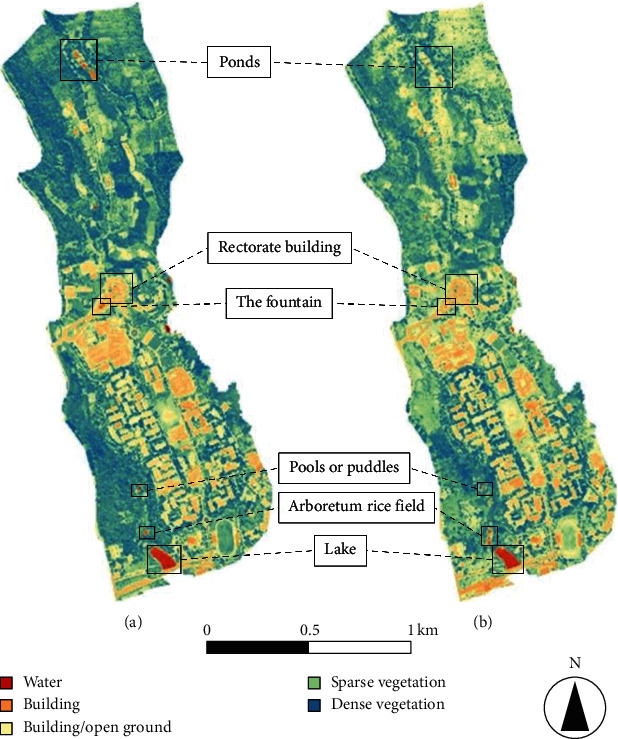
Comparison of Unpad campus land use in (a) 2015 and (b) 2017 based on NDVI analysis.

The changes in land-cover type for the Unpad campus area were determined from NDVI image maps by comparing 2015 and 2017 results (see Figure 3). We conducted an analysis of the 2015 map data and concluded that NDVI values in the range of −0.2 to 0 indicate water or wet areas on the map; this range identified pools or puddles, lakes, the arboretum rice fields, the fountains near the rectorate building, and the ponds on the Unpad's plantation. Buildings and vacant land are shown by the NDVI range 0–0.4, which are scattered throughout Unpad. Vegetation is indicated by NDVI values 0.4–1; the higher the NDVI value, the denser the vegetation that is represented. From the NDVI data, we categorized water, buildings, and bare fields as nonvegetated areas. Areas of sparse and dense vegetation we categorized as vegetated areas. We then used the same NDVI classification to analyze the 2017 data. There are obvious differences between the 2015 and 2017 maps (see Figure 3). For instance, the building/open ground areas (pale yellow) and sparse vegetation areas (green) on these image maps are more dominant than the dense vegetation (blue) areas, which means what was dense vegetation in 2015 has been reduced by 2017. In contrast, water storage (red) did not change between 2015 and 2017.

**Figure 4:**
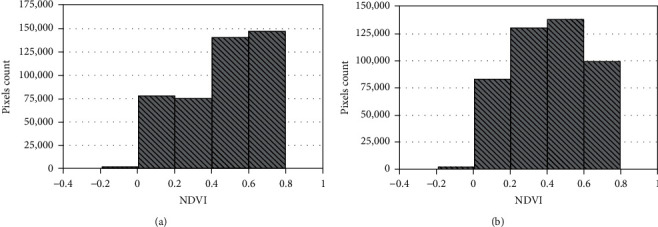
NDVI pixels histograms. (a) 2015 and (b) 2017.

To derive the percentage of each area, we ran a histogram analysis (see Figure 4); the *x*-axes show the ranges of NDVI values, and the *y*-axes show numbers of pixels that indicate that range of values. For 2015, the sum number of pixels for the vegetated areas is 288,092, whereas the sum number of pixels for nonvegetated areas is 155,196 pixels; accordingly, for 2015, the vegetated areas amount to ∼115.68 ha, or 64.99% of the ∼178 ha, and the nonvegetated areas amount to ∼62.32 ha, or 35.01% of the ∼178 ha, or ∼53.36 ha less than the vegetated areas. The results of histogram analysis for 2017 show differences compared with 2015. The dominant NDVI range for 2017 is 0.4–0.6 with a total of 138,422 pixels; the total number of pixels representing nonvegetated areas (water, buildings, and open ground) is 217,049, whereas that for the vegetated areas is 237,768 pixels. Hence, for 2017, the vegetated areas amount to ∼93.06 ha, or 52.28% of the ∼178 ha, and the nonvegetated areas amount to ∼84.94 ha, or 47.72% of the ∼178 ha, or ∼8.12 ha less than the vegetated areas.

From the histogram results, it is apparent that by 2017, the vegetation area had decreased by 12.71% since 2015, while correspondingly the nonvegetated areas had increased by 12.71% since 2015. This change was dominated by dense vegetation loss to areas of sparse vegetation or bare fields. It is estimated that this decline in vegetation is due to new building development such as Nursing Faculty and Science Technopark buildings.

### 4.2. Land Use

**Figure 5:**
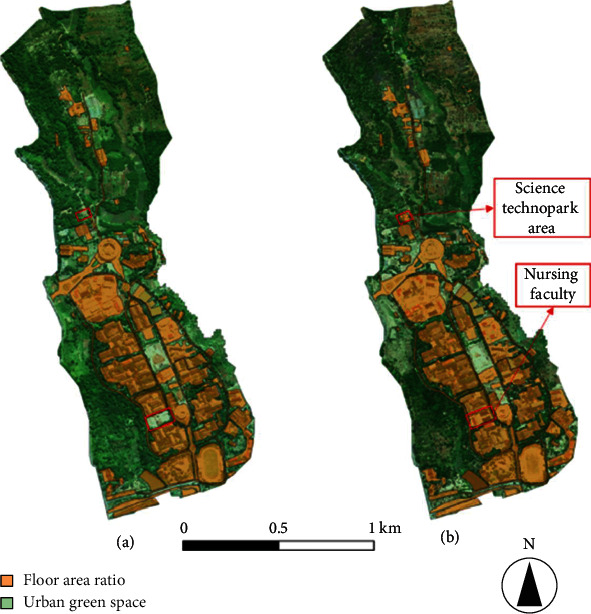
Comparison of Unpad campus land use in (a) 2015 and (b) 2017 based on NDVI analysis.

From the results of interpretation of NDVI image data and subsequent ground truthing, we derived image maps showing UGSs and FARs for 2015 and 2017 (see Figure 5).

On the land-use map produced by NDVI analysis (see Figure 5), orange areas (FAR) represent buildings and roads, and green areas (UGS) represent spaces covered by plants, including intentional plantations and the Unpad's arboretum. The results of the 2015 image-map analysis showed that the UGS areas included 124.31 ha (69.84% of the total 178 ha area), while the FAR areas amounted to 53.69 ha (30.16% of the total area), representing a difference between UGS and FAR of 70.62 ha. The north end of the study area is dominated by UGSs in the form of plantations (see Figures 1 and 5), and the south is dominated by FAR areas (mostly campus buildings). When we compared the 2015 and 2017 results of image-map analysis, we found only small differences in the areas between the two years: the UGS in 2017 amounted to 123.38 ha (69.31% of the total 178 ha), and the FAR included 54.62 ha (30.69% of the total area). Accordingly, from the two data sets, the FAR in 2017 showed an absolute increase of 0.53% compared with 2015, and the UGS correspondingly experienced an absolute decrease of 0.53% compared with 2015. The reason for the difference in FAR is the addition of two buildings on the Unpad campus: the Nursing Faculty building and Science Technopark Area (see Figure 5). According to the Perda [2], these results indicate that Unpad is still allowed to build 16.58 ha.

### 4.3. Surface-Runoff Direction

**Figure 6:**
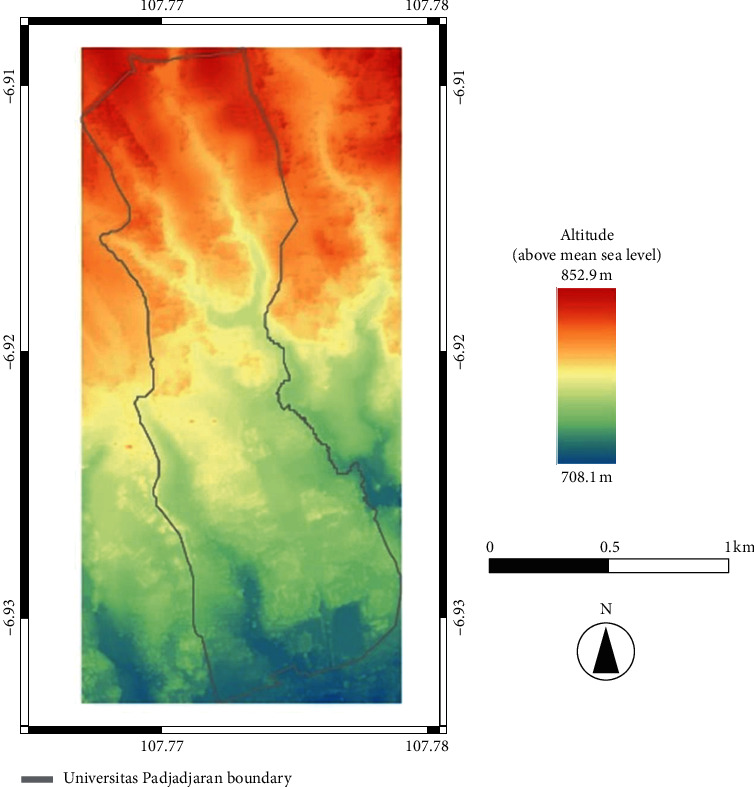
DEM of study area.

Based on the DEM map of the Unpad area (see Figure 6), the maximum altitude (red) is 852.9 m above sea level, and the minimum (blue) is 708.1 m above sea level. Accordingly, from these data, we found the difference across the entire Unpad campus to be 144.8 m and the dominant slope to be that toward the southeast.

**Figure 7:**
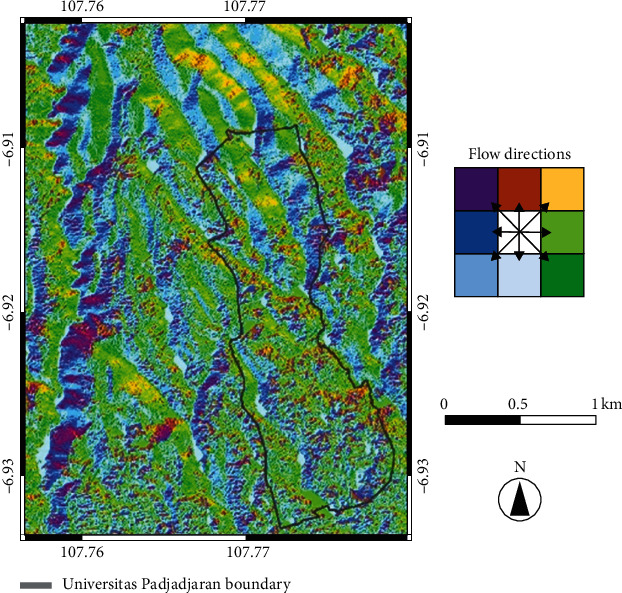
Flow-direction map of Unpad campus and vicinity.

The results of our flow-direction analysis using the D8 method (see Figure 7) indicate the possible flow direction by pixel based on the slope. The Unpad campus boundary of the Unpad area is indicated by a gray line. From this map, it is apparent that in the southern part of the Unpad campus (bounded by gray line), the pixels are dominated by green and blue, indicating that water flow is mainly in the south and toward the southeast. Our data analysis confirmed the direction of flow to be directly proportional to the direction of the dominant slope.

**Figure 8:**
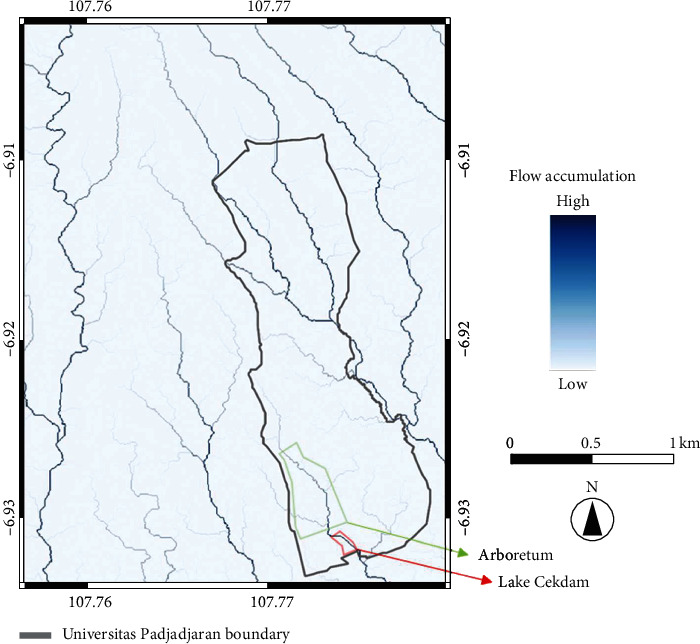
Flow-accumulation map of Unpad campus and vicinity.

From the results shown in the flow-direction map (see Figure 7), we obtained a flow-accumulation map (see Figure 8), which describes the direction of accumulated water flow. Accumulated flow (blue) is directed from lighter blue toward the darker blue. The map shows some accumulated streams that flow from the north of Unpad toward the east and southeast. This water-flow pattern can be matched to the known streamflow network around the Unpad Jatinangor campus. Another evident stream flows through the Unpad Arboretum and empties into Unpad's Lake Cekdam. To the west of the Unpad campus, other water flow through the area comes from the north or northwest. The highest accumulation occurred in the eastern part of the Unpad area and was downstream from water-flow accumulation that began to the north of Unpad.

## 5. Conclusion

From the results of this study, we found that 64.99% of the total area of the Unpad Jatinangor campus in 2015 was covered by vegetation and 35.01% of the total area was no vegetation, whereas in 2017 the land cover was 52.28% vegetated and 47.72% nonvegetated. Thus, vegetated land cover decreased by 12.71% during 2015–2017, and correspondingly, nonvegetated land cover increased by 12.71% during the same period.

We also found that in 2015, the UGS of the Unpad Jatinangor campus occupied 69.84% of the total area, while FAR areas amounted to 30.16% of the total area. For 2017, the UGS amounted to 69.31% and FAR 30.69% of the total area. Accordingly, the UGS decreased by an absolute 0.53% during 2015–2017, while FARs increased by the same amount during that period so that the area of land that can be built is 9.31% (16.58 ha). Therefore, the Unpad campus is still in compliance with the Regional Regulation (Perda) of Sumedang District number 2 of 2012 concerning regional spatial planning, which states that the ratio UGS:FAR for each business activity (including large industry) must be at least 60%:40%.

We also analyzed water-flow surface runoff and found it was dominated by flow toward the south and southeast, with accumulation in the northeastern part of Unpad through Unpad's Arboretum and into Lake Cekdam on the Unpad campus.

## Figures and Tables

**Table 1:** WorldView-2 channel type classification [23].

Channel type	Wavelength range (nm)	Spatial resolution (m)
Panchromatic	450–800	0.46
Coastal blue	400–450	1.84
Blue	450–510	1.84
Green	510–580	1.84
Yellow	585–625	1.84
Red	630–690	1.84
Red edge	705–745	1.84
Near infrared 1	770–895	1.84
Near infrared 2	860–1040	1.84

**Table 2:** Location of field inspection in Universitas Padjadjaran.

Loc. No.	Location		Photos	Actual	Maps
	*X*	*Y*			
1	107.7747243	−6.926862604	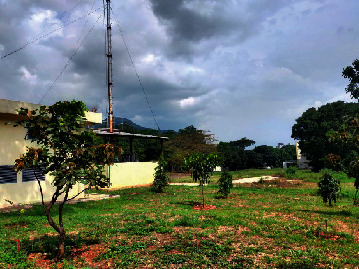	UGS	UGS
2	107.7760485	−6.926021917	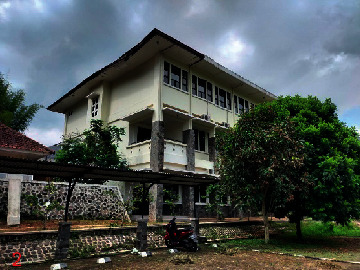	FAR	FAR
3	107.7698489	−6.909885659	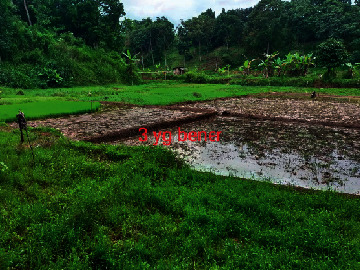	UGS	UGS
4	107.7719596	−6.931657905	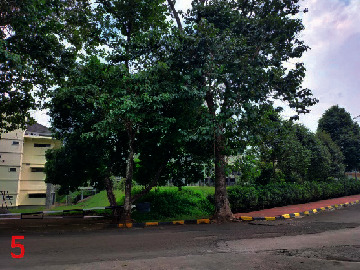	FAR	FAR
5	107.7709583	−6.921677495	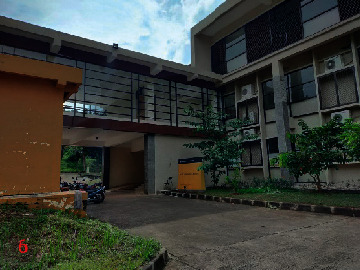	FAR	FAR
6	107.7747964	−6.924712621	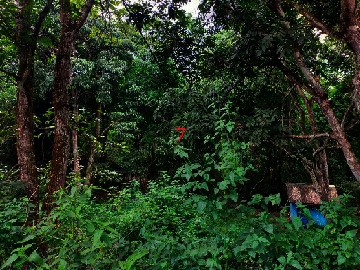	UGS	UGS
7	107.7679335	−6.911838095	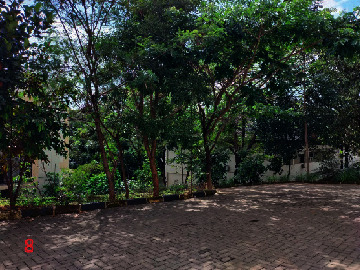	FAR	FAR
8	107.7743536	−6.928426116	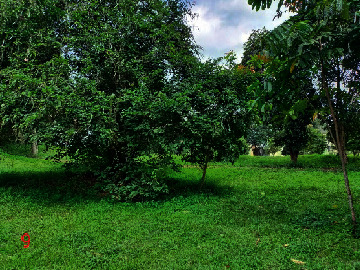	UGS	UGS
9	107.7704328	−6.92129597	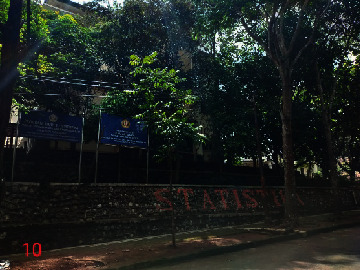	FAR	FAR
10	107.7732468	−6.924611571	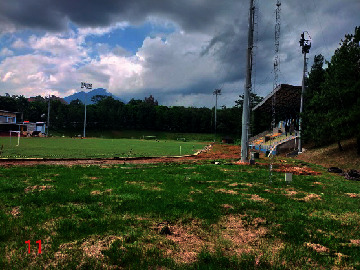	UGS	UGS
11	107.7769575	−6.930537926	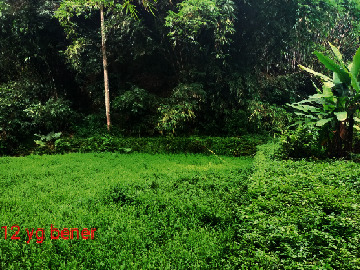	UGS	UGS
12	107.7697226	−6.909673147	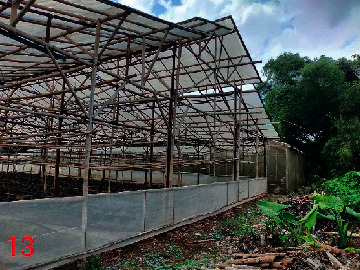	FAR	FAR
13	107.7729334	−6.919850688	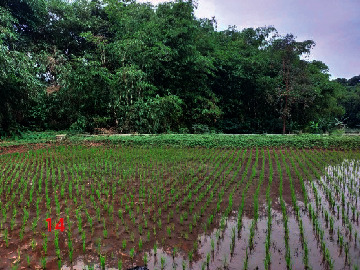	UGS	UGS
14	107.7709111	−6.917222033	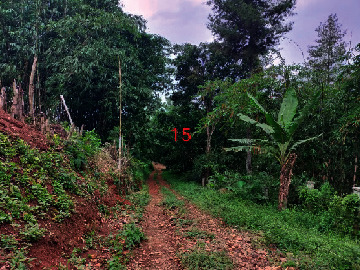	UGS	UGS
15	107.7738487	−6.918888589	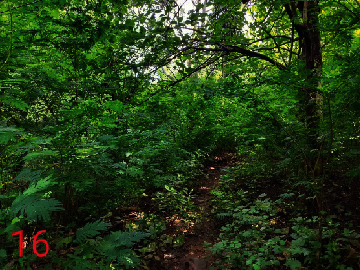	UGS	UGS
16	107.7719252	−6.927523408	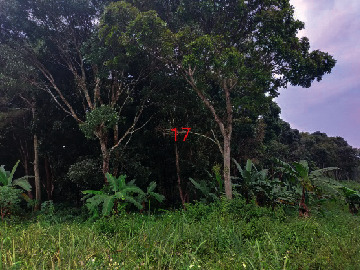	UGS	UGS
17	107.773846	−6.914943516	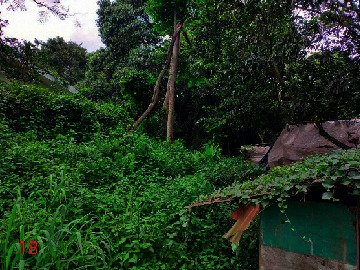	UGS	UGS
18	107.7728436	−6.931444358	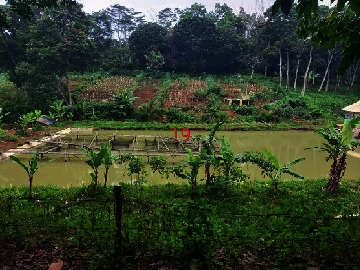	UGS	UGS
19	107.7711363	−6.911854003	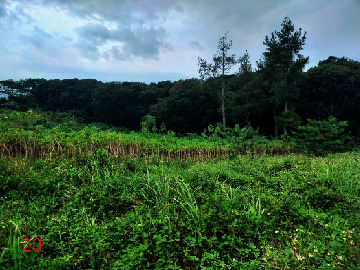	UGS	UGS
20	107.7737656	−6.914406157	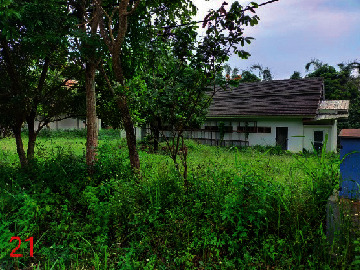	UGS	UGS
21	107.7711231	−6.915263023	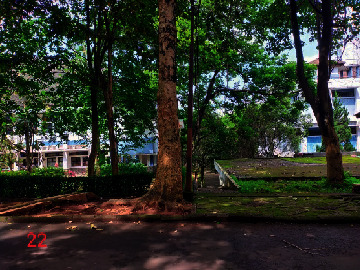	FAR	FAR
22	107.773024	−6.923773165	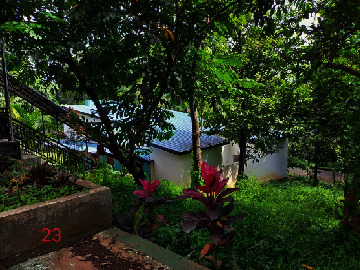	FAR	FAR
23	107.7722448	−6.926646364	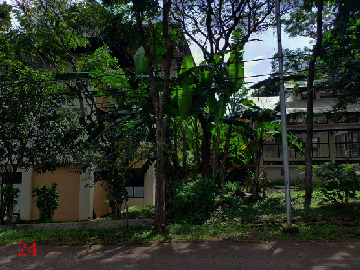	FAR	FAR
24	107.773958	−6.926164523	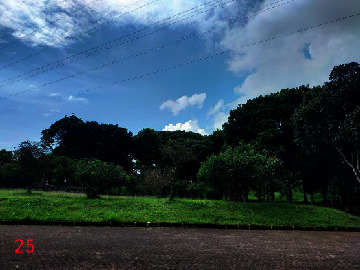	FAR	FAR

## Data Availability

Underlying data are available upon request.

## References

[1] (August 2018). 2019 QS world university rankings. https://www.universityrankings.ch/results/QS/2019?ranking=QS&year=2019&region=&q=Indonesia.

[2] (August 2018). Regional regulation of Sumedang regency number 2 of 2012. http://www.jdih.setjen.kemendagri.go.id/download.php?KPUU=11792.

[3] Muharohmah R., Putranto D. D. A. (2014). Analisis run-off sebagai dampak perubahan lahan sekitar pembangunan underpass simpang patal palembang dengan memanfaatkan teknik GIS. *Journal of Civil and Environmental Engineering*.

[4] (August 2018). Land use/land cover (LULC). https://www.nrcan.gc.ca/maps-tools-and-publications/satellite-imagery-and-air-photos/remote-sensing-tutorials/land-cover-land-use/9373.

[5] Wang J., Maduako I. N. (2018). Spatio-temporal urban growth dynamics of Lagos Metropolitan Region of Nigeria based on hybrid methods for LULC modeling and prediction. *European Journal of Remote Sensing*.

[6] Thunig H., Wolf N., Naumann S. (2010). Automated LULC classification of VHR optical satellite data in the context of urban planning. *Geographic Object-Based Image Analysis GEOBIA*.

[7] Adnan M. S. G., Abdullah A. Y. M., Dewan A., Hall J. W. (2020). The effects of changing land use and flood hazard on poverty in coastal Bangladesh. *Land Use Policy*.

[8] Rimal B., Zhang L., Keshtkar H., Sun X., Rijal S. (2018). Quantifying the spatiotemporal pattern of urban expansion and hazard and risk area identification in the Kaski District of Nepal. *Land*.

[9] Berihun M. L., Tsunekawa A., Haregeweyn N. (2019). Exploring land use/land cover changes, drivers and their implications in contrasting agro-ecological environments of Ethiopia. *Land Use Policy*.

[10] Hamoodi M. N., Corner R., Dewan A. (2019). Thermophysical behaviour of LULC surfaces and their effect on the urban thermal environment. *Journal of Spatial Science*.

[11] Chaudhuri A. S., Singh P., Rai S. C. (2018). Modelling LULC change dynamics and its impact on environment and water security: geospatial technology based assessment. *Ecology, Environment and Conservation*.

[12] Lillesand T. M., Kiefer R. W. (1979). *Remote Sensing and Image Interpretation*.

[13] Martinuzzi S., Gould W. A., Ramos González O. M. (2007). Land development, land use, and urban sprawl in Puerto Rico integrating remote sensing and population census data. *Landscape and Urban Planning*.

[14] Weng Q. (2012). Remote sensing of impervious surfaces in the urban areas: requirements, methods, and trends. *Remote Sensing of Environment*.

[15] Jhawar M., Tyagi N., Dasgupta V. (2013). Urban planning using remote sensing. *International Research Journal of Engineering Science, Technology and Innovation*.

[16] Dewan A. M., Yamaguchi Y. (2009). Land use and land cover change in Greater Dhaka, Bangladesh: using remote sensing to promote sustainable urbanization. *Applied Geography*.

[17] Xiao J., Shen Y., Ge J. (2006). Evaluating urban expansion and land use change in Shijiazhuang, China, by using GIS and remote sensing. *Landscape and Urban Planning*.

[18] Pham H. M., Yamaguchi Y., Bui T. Q. (2011). A case study on the relation between city planning and urban growth using remote sensing and spatial metrics. *Landscape and Urban Planning*.

[19] Wu Q., Li H.-q., Wang R.-s. (2006). Monitoring and predicting land use change in Beijing using remote sensing and GIS. *Landscape and Urban Planning*.

[20] Huang J., Lu X. X., Sellers J. M. (2007). A global comparative analysis of urban form: applying spatial metrics and remote sensing. *Landscape and Urban Planning*.

[21] Herold M., Scepan J., Clarke K. C. (2002). The use of remote sensing and landscape metrics to describe structures and changes in urban land uses. *Environment and Planning A: Economy and Space*.

[22] Gong C., Yu S., Joesting H., Chen J. (2013). Determining socioeconomic drivers of urban forest fragmentation with historical remote sensing images. *Landscape and Urban Planning*.

[23] (October 2018). The benefits of the 8 spectral bands of WorldView-2. https://dg-cms-uploads-production.s3.amazonaws.com/uploads/document/file/35/DG-8SPECTRAL-WP_0.pdf.

[24] Masek J. G., Lindsay F. E., Goward S. N. (2000). Dynamics of urban growth in the Washington DC metropolitan area, 1973-1996, from Landsat observations. *International Journal of Remote Sensing*.

[25] Elshehaby A. R., Taha L. G. E.-D. (2009). A new expert system module for Building detection in urban areas using spectral information and LIDAR data. *Applied Geomatics*.

[26] Mugiraneza T., Nascetti A., Ban Y. (2019). WorldView-2 data for hierarchical object-based urban land cover classification in kigali: integrating rule-based approach with urban density and greenness indices. *Remote Sensing*.

[27] Bacher U., Mayer H. (2005). Automatic road extraction from multispectral high resolution satellite images. *Proceedings of CMRT05*.

[28] Ribeiro B. M. G., Fonseca L. M. Urban land cover classification using WorldView-2 images and C4. 5 algorithm.

[29] Jombo S., Adam E., Byrne M. J., Newete S. W. (2020). Evaluating the capability of WorldView-2 imagery for mapping alien tree species in a heterogeneous urban environment. *Cogent Social Sciences*.

[30] Tempfli K. DTM and differential modelling.

[31] (October 2018). Landcover according to SNI. http://www.big.go.id/assets/download/sni/SNI/15.%20SNI%207645-2010%20Klasifikasi%20penutup%20lahan.pdf.

[32] Wang L., Liu H. (2006). An efficient method for identifying and filling surface depressions in digital elevation models for hydrologic analysis and modelling. *International Journal of Geographical Information Science*.

[33] (August 2018). Regulation of the minister of public works number 06/PRT/M/2007 concerning general guidelines for building and environmental planning. http://nawasis.org/portal/digilib/read/peraturan-menteri-pekerjaan-umum-nomor-06-prt-m-2007-tentang-pedoman-umum-rencana-tata-bangunan-dan-lingkungan/51333.

[34] Lindgren D. T. (1985). *Land Use Planning and Remote Sensing*.

[35] (August 2018). Unpad area. https://www.google.com/earth/.

[36] Tucker C. J. (1979). Red and photographic infrared linear combinations for monitoring vegetation. *Remote Sensing of Environment*.

[37] Arnanto A. (2013). Pemanfaatan transformasi normalized difference vegetation index (NDVI) citra Landsat TM untuk zonasi vegetasi di Lereng merapi bagian selatan. *Geomedia*.

[38] Bellón B., Bégué A., Lo Seen D., de Almeida C., Simões M. (2017). A remote sensing approach for regional-scale mapping of agricultural land-use Systems based on NDVI time series. *Remote Sensing*.

[39] Fathizad H., Tazeh M., Kalantari S., Shojaei S. (2017). The investigation of spatiotemporal variations of land surface temperature based on land use changes using NDVI in southwest of Iran. *Journal of African Earth Sciences*.

[40] Baeza S., Paruelo J. M. (2020). Land use/land cover change (2000-2014) in the rio de la Plata grasslands: an analysis based on MODIS NDVI time series. *Remote Sensing*.

[41] Zaidi S. M., Akbari A., Abu Samah A., Kong N., Gisen J. (2017). Landsat-5 time series analysis for land use/land cover change detection using NDVI and semi-supervised classification techniques. *Polish Journal of Environmental Studies*.

[42] Hu Y., Dong Y., Batunacun (2018). An automatic approach for land-change detection and land updates based on integrated NDVI timing analysis and the CVAPS method with GEE support. *ISPRS Journal of Photogrammetry and Remote Sensing*.

[43] Reed B. C., Brown J. F., VanderZee D., Loveland T. R., Merchant J. W., Ohlen D. O. (1994). Measuring phenological variability from satellite imagery. *Journal of Vegetation Science*.

[44] Duarte L., Teodoro A. C., Monteiro A. T., Cunha M., Gonçalves H. (2018). QPhenoMetrics: an open source software application to assess vegetation phenology metrics. *Computers and Electronics in Agriculture*.

[45] Jensen J. R. (2000). Active and passive microwave, and LIDAR remote sensing. *Remote Sensing of the Environment: An Earth Resource Perspective*.

[46] Wang G., Nunez M. (1990). A new method of estimating path radiance for band ratio applications. *International Journal of Remote Sensing*.

